# Supramolecular Nanostructures Based on Cyclodextrin and Poly(ethylene oxide): Syntheses, Structural Characterizations and Applications for Drug Delivery

**DOI:** 10.3390/polym8050198

**Published:** 2016-05-17

**Authors:** Yue Zheng, Ian W. Wyman

**Affiliations:** 1 Department of internal medicine, The First Hospital in Qinhuangdao Affiliated to Hebei Medical University, Qinhuangdao 066004, China; 2 Department of Chemistry, Queen’s University, Kingston, ON K7L 3N6, Canada; 3iww@queensu.ca

**Keywords:** supramolecular polymers, cyclodextrins (CDs), poly(ethylene oxide), syntheses, structure, drug delivery

## Abstract

Cyclodextrins (CDs) have been extensively studied as drug delivery carriers through host–guest interactions. CD-based poly(pseudo)rotaxanes, which are composed of one or more CD rings threading on the polymer chain with or without bulky groups (or stoppers), have attracted great interest in the development of supramolecular biomaterials. Poly(ethylene oxide) (PEO) is a water-soluble, biocompatible polymer. Depending on the molecular weight, PEO can be used as a plasticizer or as a toughening agent. Moreover, the hydrogels of PEO are also extensively studied because of their outstanding characteristics in biological drug delivery systems. These biomaterials based on CD and PEO for controlled drug delivery have received increasing attention in recent years. In this review, we summarize the recent progress in supramolecular architectures, focusing on poly(pseudo)rotaxanes, vesicles and supramolecular hydrogels based on CDs and PEO for drug delivery. Particular focus will be devoted to the structures and properties of supramolecular copolymers based on these materials as well as their use for the design and synthesis of supramolecular hydrogels. Moreover, the various applications of drug delivery techniques such as drug absorption, controlled release and drug targeting based CD/PEO supramolecular complexes, are also discussed.

## 1. Introduction

Some tissues and biological barriers [1,2,3,4,5,6,7] within our bodies prevent foreign materials, such as drugs, pollutants, and viruses, from accessing the important organs [8,9]. Although these protections play key roles in allowing the human body to remain healthy, they can also present a significant challenge with regard to the development of for the drug delivery systems. Supramolecular hosts particularly those based on macrocycles, have attracted much attention because they provide the precursors for novel biomedical materials and can help to further our understanding of natural supramolecular self-assembly.

Cyclodextrins (CDs) are macrocyclic oligosaccharides, containing six, seven and eight glucose units, which are denoted as α-, β-, and γ-CDs [10,11], respectively. CDs are able to form inclusion complexes with different molecular weight compounds [12,13,14,15,16], due to their possession of a hydrophobic central cavity and a hydrophilic outer surface. In order to form inclusion complexes, it is necessary that the guest molecules will have a compatible size that allows them to fit within the cavities of the CDs and that they will have sufficiently strong binding affinities to facilitate complexation. Furthermore, factors influencing the formation of the inclusion complexes can also include the conformational energy [17], vander Waals interactions [18], and the formation of hydrogen bonds. Usually, complex formation results in a dynamic equilibrium of the complex, CDs and the guest, which is characterized by the association constant, which is expressed as follows [19]:

(1) $$Ka=\frac{\left[G/CD\right]}{\left[G]\times [CD\right]}$$

where [G], [CD] and [G/CD] represent the concentrations of the guest, CDs and the host–guest complex, respectively. The *K*_a_ will determine the strength of the binding, and in the case of a large binding affinity, the formation of the host–guest complex is highly favored.

CDs have been extensively used as drug delivery carriers through host–guest interactions, due to their merits in increasing drug solubility [20,21], controlling drug release profiles [22], alleviating systemic toxicity [23], and improving the permeability of biological barriers [24]. Poly(ethylene oxide)(PEO) has been widely applied in biomedical fields due to its solubility in water and its biocompatibility [25,26]. Among PEO chains, those with low molecular weights exhibit its excellent chain flexibility, while those with high molecular weights possess high mechanical strength and toughness. Moreover, the hydrogels of PEO are also extensively researched for their promising potential as drug delivery vehicles [27]. Recently, CD/PEO-based poly(pseudo)rotaxanes, whose inclusion complexes were composed of multiple CD rings that were threaded onto a polymer chain with (or without) bulky end-caps, vesicles, or supramolecular hydrogels, have led to an exciting interesting developments in the biomaterials field.

While some of the PEO-based polymers described in this review will include homopolymers that consist solely of a PEO chain, many of the polymers described are block copolymers. Block copolymers consist of two or more distinct chains (or blocks) that are covalently linked together [28]. Because the two blocks are often incompatible with one another, block copolymers tend to undergo phase segregation, thus potentially forming a wide range of nanostructures with intricate morphologies.

In solution, amphiphilic diblock copolymers consisting of a water-soluble and a poorly soluble hydrophobic block can aggregate to form micelles when their concentration reaches the critical micelle concentration (CMC). In an aqueous solution, the hydrophobic chains will tend to collapse, while the hydrophilic chain will be solvated and somewhat stretched, extending into solution. At low concentrations, the block copolymers bearing collapsed and solvated chains may exist as individual micelles or unimers. When the CMC is exceeded, these unimers may aggregate to form micelles, which consist of an insoluble core domain that is surrounded by a soluble “corona” or “shell”, and hence these micelles are known as core–shell micelles. While spherical core–shell micelles are the most common, a wide range of micellar morphologies have been observed since Eisenberg and coworker’s discovery of non-spherical morphologies in the 1990s [29,30]. The most common micellar morphologies exhibited by diblock copolymers include spherical and cylindrical micelles, as well as vesicles. A number of factors can influence the micellar morphology, including the volume ratio of the hydrophobic and hydrophilic blocks, the nature of the solvent, the presence of salt, the rigidity of the individual blocks, and various other factors [31]. The range and complexity of the micellar morphologies becomes even more complex among block copolymers containing three or more blocks. In this review, we summarize the recent progress regarding the drug delivery applications ofsupramolecular architectures based on CD and PEO from 2008 to present. Particular focus will be devoted toward the structures and properties, the design and synthesis of poly(pseudo)rotaxanes, vesicles, and supramolecular hydrogels. Moreover, the various applications of drug delivery techniques such as drug absorption, controlled release and drug targeting based on CD/PEO supramolecular complexes, are also discussed.

## 2. Supramolecular Structure and Properties of CD/PEO-Based Poly(pseudo)rotaxanes

The first report on the synthesis of CD-based poly(pseudo)rotaxanes was described by Harada which was based on the block of poly(ethyleneoxide) (PEO) and poly(propylene oxide)(PPO) [32,33]. The supramolecule composed of multiple α-CD rings threaded on the PEO chain and trapped by end-capping the chain with bulky end groups. In the case of polyrotaxanes(PRs), the CDs are fixed onto certain regions of the polymer chains, typically due to the presence of bulky functionalities that restrict their migration. However, in the case of poly-pseudorotaxanes(PPRs), CDs can move along or off the polymer backbone because they lack stoppers [34]. Poly(pseudo)rotaxanes, including the polymers of PEO, have been employed for the encapsulation and delivery of drugs [35,36,37,38,39], because their molecular structure and properties can be finely tuned by modulating the length of the PEO chain [40,41,42]. In controlling the formation of the inclusion complex, the cross-sectional regions of the polymer chains and the diameters of the cavities of CDs were considered the important factor. Recently, size-correlated factors have been given growing attention, because CDs may recognize and selectively complex with different blocks, which would help with the design and synthesis of novel biomaterials for potential drug delivery applications.

### 2.1. CD/PEO-b-PPO-b-PEO

**Figure 1:**
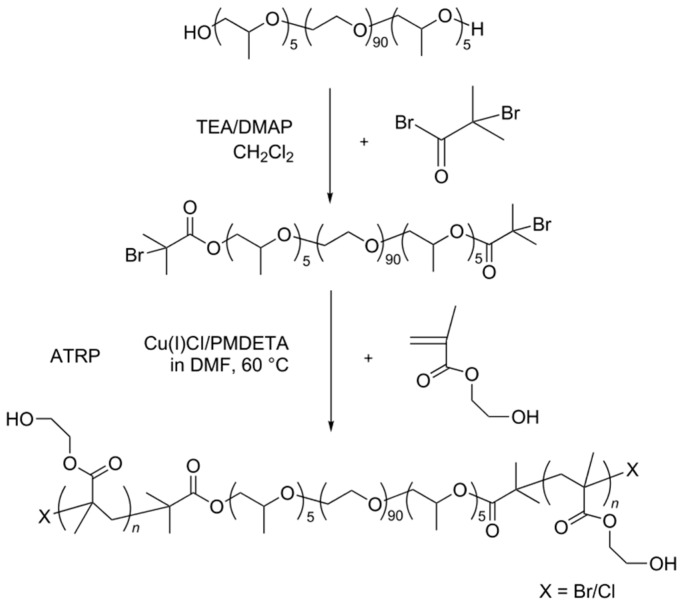
Synthetic pathway toward a pentablock copolymer. This scheme was adapted with permission from [43] Copyright (2014) Beilstein-Institut.

**Figure 2:**
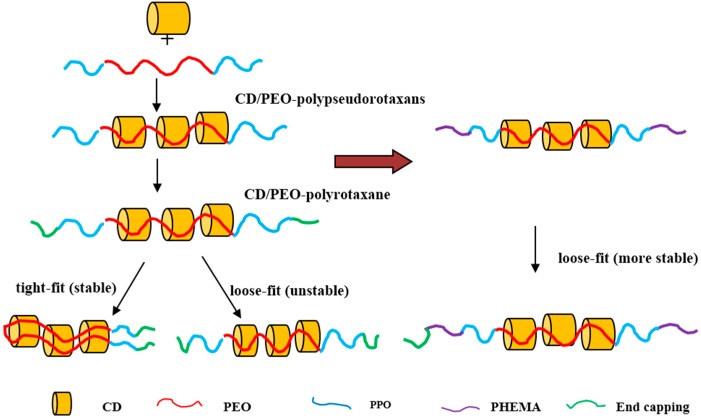
Schematic depiction of the self-assembly of CD/PPO-*b*-PEO-*b*-PPO and CD/PHEMA-*b*-PPO-*b*-PEO-*b*-PPO-*b*-PHEMA poly(pseudo)rotaxanes. The diagram was adapted and revised from reference [43]. Copyright (2014) Beilstein-Institut, and adapted with permission from reference [48]. Copyright (2010) American Chemical Society.

CDs can act as host molecules to linear polymeric guest molecules, because the dimensions of CDs may vary depending on the number of glucose units within their macrocyclic ring. In the cases involving γ-CDs either single or double polymer chains may be threaded through the hydrophobic cavities of the CDs depending on the influence of the solution environment and the intermolecular interactions involved. Although the self-assembly behavior of CD-based poly(pseudo)rotaxanes is mostly determined by the cross-sectional area of the incoming polymer chain and the cavity size of the CDs, for γ-CDs, their inclusion behavior depends on the solution, guest molecules, and especially the number of glucose units. Feng *et al.* [43] reported the capping of a triblock copolymer PPO-*b*-PEO-*b*-PPO by poly(2-hydroxyethyl-methacrylate) (PHEMA)-blocks via atom transfer radical polymerization (ATRP) in DMF to thus yield a pentablock copolymer PHEMA-*b*-PPO-*b*-PEO-*b*-PPO-*b*-PHEMA allowed this copolymer to self-assemble with γ-CDs in aqueous solution [43], whose inclusions showed the single-chain. The synthetic pathway towards this pentablock copolymer is shown in Figure 1. In this synthesis, Cu(I)Cl/PMDETA was chosen as the catalyst and DMF as solvent for the ATRP of 2-hydroxyethyl methacrylate (HEMA). BrPEPBr, as shown in Figure 1, was designated as the macroinitiator and PEP26M was the pentablock copolymer. The PEP18CD(feed molar ratio of BrPEPBr/γ-CD = 1:18) was self-assembled by BrPEPBr and γ-CDs, and PEP26MnCD were composed of γ-CDs with PHEMA-*b*-PPO-*b*-PEO-*b*-PPO-*b*-PHEMA(n represents the feed molar ratio of γ-CD to PEP26M). The passage of time led to a diverse range of structures, such as single-chain stranded or loose-fitting (Figure 2) complexes, as was confirmed by ^1^H NMR, WXRD, DSC, TGA, ^13^C CP/MAS NMR and FTIR analysis. Single-chain stranded γ-CD-based PPRs or PRs, in comparison with the typical double-chain stranded PPRs, such as those reported by Harada *et al.* [44], showed no characteristic channel-type crystal structures. In those, the inclusion complexes expressed a superior ability to undergo deformation and to adapt to the external conditions so that the γ-CDs could readily move along the polymer chains [45,46,47,48].

CD derivatives can also influence the characters, both the inclusions and the poly(pseudo)rotaxanes. The complexation behavior off our β-CD derivatives, including DIMEB (heptakis(2,6-di-*O*-methyl)-β-CD), TRIMEB (heptakis(2,3,6-tri-*O*-methyl)-β-CD), HEBCD(hydroxyethyl-β-CD), and HPBCD(hydroxypropyl-β-CD), with micelles of Pluronic F127(PEO_100_-*b*-PPO_65_-*b*-PEO_100_), were investigated and the disassembly behavior between the CDs and the micelles was reported [49]. The interactions involving the CD derivatives and inclusions could lead to the disassembly, which resulted from the methyl groups in position 6 of the glucopyranose rings of DIMEB and TRIMEB with the inclusions, such as PPO. Based on this study, their research on the competitive interactions of CDs and drugs with PEO-PPO-PEO by the assembly and disassembly of micelles was researched [50].

Among the β-CD derivatives, such as heptakis(2,3,6-tri-*O*-methyl)-β-CD, 2-hydroxyethyl-β-CD and 2-hydroxypropyl-β-CD, hep2,6-β-CD would lead to the disassembly of the Pluronicmicelles, due to the quantity of free hydroxyl groups on the β-CD which altered its hydrophobicity. More interestingly, they discussed the competitive interactions, between β-CD, the Pluronic micelle and the drug. On the one hand, the presence of hep2,6-β-CD in solution would break-up the micelles. However, on the other hand, the presence of the drug in the micelles would help to hold them together via the hydrophobic effect. Therefore, in this three-component competitive system, the drug would form complexes with β-CD at the expense of the formation of pseudopolyrotaxanes between β-CD and the PPO segment. This research described the changes in the micellar structures occurring upon encapsulation of a drug, and proposed opening-up micelles based on β-CD/pseudopolyrotaxanes, as a handle to trigger or control the release of a drug.

The intermolecular hydrogen bonding, as the driving force for the self-assembly of CDs with polymer chains between the neighboring CDs, can only be destroyed by a few solvents: *N*,*N*-dimethylformamide (DMF), dimethyl sulfoxide (DMSO), *N*,*N*-dimethylacetamide (DMAc), and ionic liquids [51] can disrupt the hydrogen bonding. PRs with tunnel-type or tunnel-like structures would be in a dispersed-state with the destructive solvents. Feng *et al.* [51] synthesized a series of polyrotaxane-based β-CDs and Pluronic F127 with poly(*N*-isopropylacrylamide) (NIPAAm)as end-stoppers via atom transfer radical polymerization (ATRP) in an a queous *N*-isopropylacrylamide solution. The as-prepared polyrotaxane exhibited not only solvent-sensitive and thermoresponsive behavior, but also exhibited fascinating aggregation structures. The polyrotaxane did not have the characteristic tunnel-type crystal structure, but instead exhibited a dispersed-state structure, after it had been precipitated from DMF with anhydrous ether. In these copolymers, the distribution of β-CD along the Pluronic F127 chain was loosened. However, after the system had been subjected to incubation in water and freeze-drying treatment, the structure of the polyrotaxane-based β-CDs and Pluronic F127 formed a tunnel-like crystal. Under the treatment of solvent, the interconversion of two structures would happen. In addition, the movements of the entrapped CDs in the solid state showed an opposite movement tendency in the dispersed and aggregated states, which rendered them thermoresponsive. These findings would inspire the preparation of potential thermoresponsive polymeric materials by the simple solvent treatments.

The structures of CD derivatives influenced the properties of the inclusion complexes (ICs). For example, this was demonstrated by a physico-chemical study for the copolymer based on PEO-*b*-PPO-*b*-PEO and CDs [48]. The CDs, including native and hydroxypropyl CDs with different cavity sizes were chosen to investigate the role of the molecular weight in influencing the properties of the inclusion complexes (ICs). A channel structure was shown when the native was adopted in the complex; however, necklace-like structures were generated if alkylated-CDs decorated the copolymer, when the conditions, such the hydrophilic/hydrophobic ratio and the hydrophilicity were kept constant based on volume, DSC, and FTIR characterization. Lazarra *et al.* [52] had found that the inclusion complexes were in anamorphous state and their thermal stability depended on the nature of both the CD and the copolymer based on volume, DSC, and FTIR investigation.

The formation of supramolecular complexes between CDs and PEO-*b*-PPO-*b*-PEO has motivated many researchers to investigate their fascinating self-assembly behavior. In addition, the solvent-sensitive or thermoresponsive properties, as well as the PPRs or PRs as inclusions consisting of either one or two threaded chains characteristic crystal structure, and the influence of CD and their derivatives, have pushed the applications of this kind of novel biomaterial for drug delivery.

### 2.2. CD/PEO-b-PDEAEMA

Polymerswith functional properties of coordination with the metal ions have become powerful tools as the “special medicine” carrier for cancer diagnosis and radiotherapy. When the environment changes, such as pH, temperature, light and ion concentration, the block copolymers, with the appropriate structural design, would form the micelles in pure water. The transition metal ions ^67^Cu (half-life 2.58 days) and ^90^Y(III) (half-life 2.67 days) can be loaded in the micelles for use in radiotherapy.Poly(2-(diethylamino)ethyl methacrylate) (PDEAEMA) is apt to coordinate with metal ions and be a pH-responsive block, which is a hydrophilic block at low pH, and becomes a hydrophobic block at higher pH. Therefore, the diblock copolymer PEO_45_-*b*-PDEAEMA_45_was synthesized as the carrier of transition metal ions Y(III) and Cu(II)via anionic polymerization [53]. Due to the differences in the solubility between the different blocks, this copolymer formed unique micelle structures by self-assembly in neutral and alkaline aqueous media. The soluble corona was formed by the PEO block, while the insoluble PDEAEMA block formed the micellar core. Furthermore, the cores of these micelles could be loaded with metal ions, such as Y(III) and Cu(II), due to the PDEAEMA block’s ability to coordinate with metal ions. More importantly, the Y(III) loaded micelles could form supramolecular gels *in situ*, by the simple addition of α-CDs. These materials may have strong potential as an internal radiotherapy system, replacing traditional chelating ligands as carriers.

### 2.3. CD/PEO

**Figure 3:**
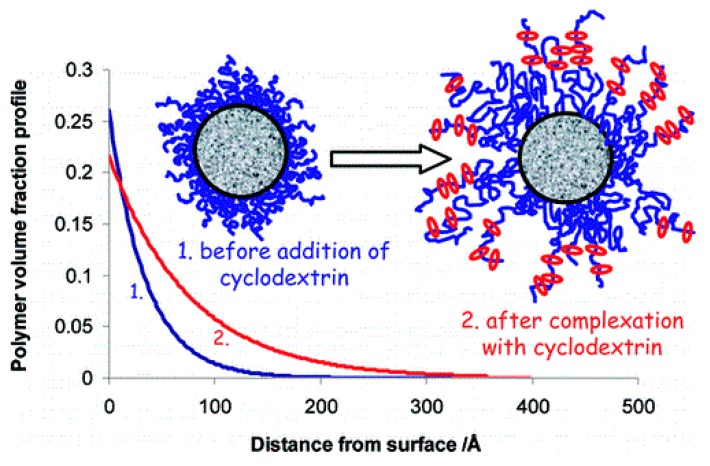
Diagram depicting of the effects of adding cyclodextrin on the polymer profile. This image was adapted with permission from [57]. Copyright (2008) American Chemical Society.

Because the adsorbed polymers can increase the stabilization of colloidal particles, many groups have focused on the research of additions [54,55,56]. Dreiss *et al.* [57] discussed the interactions between grafted polymer layers and CDs via small angle neutron scattering measurements. They found that the addition of CDs affected the polymer structure. In particular, the PEO chains were extended into the bulk and this behavior was attributed to the formation of a pseudopolyrotaxane based on CD/PEO. Among these mixtures that were investigated with different CD homologs, the most significant increase in the layer thickness was observed in cases involving α-CD constituents. The signal from the layers was fitted to a double-exponential volume fraction profile, as shown in Figure 3, which was attributed to the stronger complexation between PEO and α-CD.

**Figure 4:**
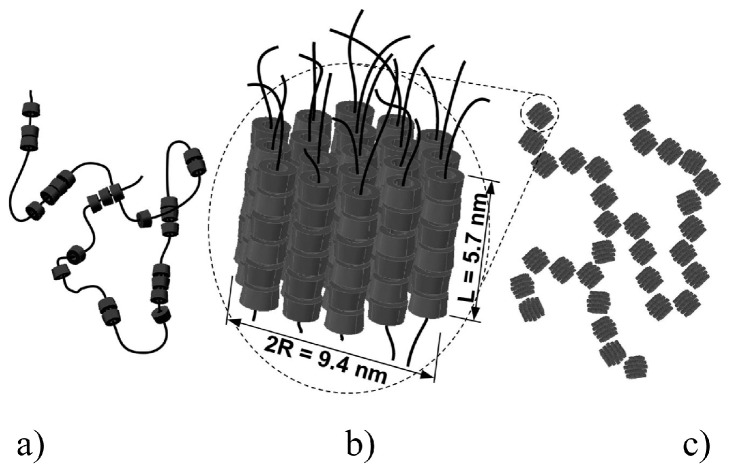
(**a**) A PPR molecule with α-CD and naked PEO segments; (**b**) α-CD-based PPR molecule; and (**c**) α-CD-based PPR molecule in a Gaussian way. This image was adapted with permission from [58]. Copyright (2009) American Chemical Society.

PPRs (α-CD/PEO-based polyrotaxanes) were obtained at low temperature (5 °C) at good yield of the synthesis in water. However, it is difficult to chemically modify the formed physical gel at this temperature. Schlatter *et al.* [58] reported a specific behavior at 30 °C of α-CD/PEO-based pseudopolyrotaxanes in water and discussed the kinetics of formation and self-organization. Usually, for the liquid mixture of α-CD/PEO at 70 °C, a white physical gel of PPRs appeared with time at a lower temperature by quenching. At 30 °C, a physical gel of PPRs formed more slowly and the process could be described as a two-step phenomenon: In the first step, the PPR molecules were poorly soluble in water, although α-CDs had threaded onto the PEO chains. During the second step, the system underwent structural reorganization and the amount of precipitated domains increased. The schematic is shown in Figure 4. Their work on the PPR synthesis at 30 °C provides valuable new insight regarding PPR chemical modification for drug delivery.

### 2.4. CD/PEO-b-POO-b-PEO

Luis *et al.* [59] reported the competitive displacement between the drugs being incorporated, CD and PEO-*b*-POO-*b*-PEO by polypseudorotaxane formation. The studies on the solubility, drug release and competitive displacement, were one-dimensional experiments using^1^HNMR and 2D Rotating-frame Overhauser Effect Spectroscopy (ROESY). They concluded that stability of the drug–CD complexes and the CD concentration in the bulk solution had significantly influenced the release rate and the formation of polypseudorotaxanes. In particular, with regard to the solubilization capability, the formation of polypseudorotaxanes disrupted the formation of the complexes inclusion of CD–drug, which reduced the solubilization, but the micellization of PF127 in complexes a queous solutions compensated the ability of solutions. The ability of PF127 to displace guest molecules from the CD cavities can effectively modify the release properties of the aqueous drug–CDs complexes, which would provide valuable opportunities for new drug delivery systems based on the formation of polypseudorotaxanes.

### 2.5. CD/PPO-b-PEO-b-PPO

Isasi *et al.* [60] reported the influence of the different kind of CDs on the nature of the resultant polypseudorotaxane structures. A series of supramolecular polymers were prepared based on the Pluronic10R5 (PPO_8_-*b*-PEO_22_-*b*-PPO_8_) with β- and α-CD, which had different structures and properties. When α-CD was combined with Pluronic 10R5, they became localized on the PEO blocks of 10R5, while the hydrophobic PPO blocks aggregated in aqueous media, due to the hydrophobic effect. Consequently, the viscosity of the system increased, thus leading to the formation of fluid gels. The CD/copolymer ratio was the key factor in this kinetics process. Higher CD/copolymer ratios accelerated the onset of cloudiness and promoted coalescence appeared. In other cases, the entire process would last several days. By varying the ratio between 10R5 and CD, the viscosity could be tuned. Meanwhile, when 10R5 was mixed with β-CD, these larger CDs favored the PPO block. These polypseudorotaxanes formed white precipitates with crystalline structures.

New polyrotaxanes, PPO-polyrotaxane-PPO, were synthesized based on PPO-*b*-PEO-*b*-PPO and α-CD with two 2,4-dinitrophenyl groups as terminal group [61]. During the formation of new polymers, α-CDs covered the PEO segments of the PPO-*b*-PEO-*b*-PPO triblock copolymer after they had slid over the PPO segments. They also found that the PEO block length determined the numbers of α-CD rings that would become threaded onto the polymer chain. In addition, the introduction of α-CD enhanced the thermal stability of the new polyrotaxanes.

Recently, many reports have focused on the stimuli-responsive properties of polyrotaxanes, due to their reversible changes in response to external conditions, such as the pH or temperature [62,63,64]. Li *et al.* [65] investigated a cationic polyrotaxane based on a pentaethylenehexamine(PEHA)-grafted α-CD and PPO-*b*-PEO-*b*-PPO triblock copolymer that exhibited thermoresponsive behavior. The cationic polyrotaxane was composed of six α-CD rings that were treaded onto one polymeric chain and an average of 4.8 PEHA chains were grafted onto each α-CD ring. In the synthesis, the cationic polyrotaxane could form multi-molecular micelles with increasing concentration and temperature. Among the parameters influencing the micellization, including the standard free energy (∆*G*°), enthalpy (∆*H*°), and entropy (∆*S*°), entropy was found to be the dominant factor driving the micellization.

## 3. Vesicles/Polymersomes of CD/PEO-Based Linear Block Copolymers for Drug Delivery

Polymeric vesicles have attracted significant attention due to their hollow structures and their strong potential for drug delivery applications [66,67,68,69,70]. Among the block copolymers, amphiphilic copolymers are the most commonly used [71,72,73,74,75] building blocks of polymersomes. In aqueous solution, the insoluble hydrophobic block typically collapses from solution to form the polymersome wall, while the hydrophilic blocks extend into solution. In comparison with spherical cells, the insoluble block of a vesicle-forming diblock copolymer generally has a larger volume ratio. Although many groups investigated the building blocks for the preparation of polymeric vesicles with amphiphilic block copolymers [76,77,78], there are still many challenges regarding the preparation of these nanostructures that will need to be addressed if these self-assembly techniques are to become widely applied in drug delivery, such as their complicated preparation processes [79,80], the need for organic solvents, and so on.

### 3.1. CD/PEO-b-PMPC

Ji *et al.* [81] prepared biocompatible polymer vesicles based on α-CDs and poly(ethylene oxide)-*b*-poly(2-methacryloyloxyethyl phosphorylcholine) (PEO-*b*-PMPC) in aqueous media. In this case, the polymer vesicles resulted in the self-assembly of polyrotaxanes based on α-CD and PEO-*b*-PMPC. They discussed the delivery and release efficiency of the drug by loading hydrophilic doxorubicin (DOX·HCl) into these vesicles. Experimental results showed this complex had a lower cytotoxicity than drug-loaded vesicles that did not incorporate α-CDs and that it enhanced the delivery and the release of drug to the targeted site.

### 3.2. CD/PEO-b-PNIPAAm

Ji *et al.* [82] investigated the formation of micelles and reverse micelles based on CD/PEO-*b*-PNIPAAm polyrotaxanes, with particular emphasis on the effect of α-CD and the temperature on the micellar structures. They synthesized poly(ethylene oxide)-*b*-poly(*N*-isopropylacrylamide)(PEO-*b*-PNIPAAm) via atom transfer radical polymerization(ATRP). When the temperature was 40 °C, the copolymer underwent self-assembly to form micelles in which the PEO block acted as the corona and the PNIPAAm block formed the micellar core. However, when the temperature was decreased to 25 °C, the PNIPAAm-core micelles underwent disassembly. Moreover, they found that adding the α-CD into the mixture of the PNIPAAm-core micelles and their unimers led to the formation of reverse micelles, in which the PEO block formed the core and the PNIPAAm block acted as the corona. This resulted in micelles with the formation of “channel-type” crystallites induced by the polyrotaxanes of PEO/α-CDs. The construction of micelles and reverse micelles may provide a facile approach for the delivery of stimuli-responsive drugs.

### 3.3. CD/HBPO-Star-PEO

**Figure 5:**
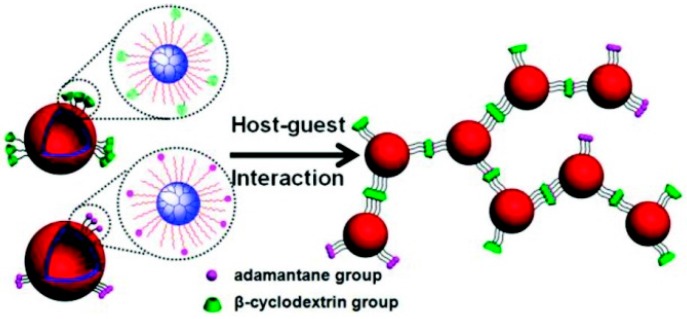
Schematic diagram of cytomimetic large-scale vesicle aggregation and fusion based on host–guest interaction was adapted with permission from [83]. Copyright (2012) American Chemical Society.

Zhou *et al.* [83] investigated their studies on the aggregation and fusion of vesicles based on CD/HBPO-star-PEO [HBPO = hyperbranched poly(3-ethyl-3-oxetanemethanol); PEO = poly(ethylene oxide)] used as a drug carrier, by the interaction between the host and guest (Figure 5). First, they synthesized three polymers as the building blocks: HBPO-star-PEO, a damantane-functionalized branched polymersomes (Ada-BPs), and β-CD-functionalized branched polymersomes (CD-BPs). Second, cell-sized polymer vesicles were prepared by the host–guest recognition interactions between the adamantine groups and β-CDs on the branched polymersomes by simply mixing the Ada-BPs and CD-BPs together. In their experiment, large-scale vesicle aggregation and fusion resulted from the strong intervesicular interaction of Ada-BPs and CD-BPs. These novel hierarchical polymer vesicles may lead to applications, in the development of tissue materials.

## 4. Supramolecular Hydrogels Based on CDs and PEO for Drug Delivery

**Figure 6:**
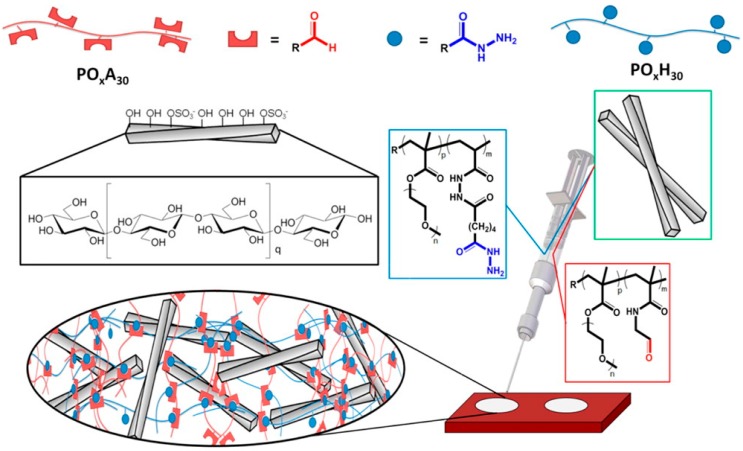
Synthesis schematic of poly(oligoethylene glycol methacrylate) injectable hydrogel through control of physical and chemical cross-linking. Reprinted with permission from [89]. Copyright (2016) American Chemical Society.

Hydrogels have attracted much attention as potential drug delivery systems due to their hydrophilicity, biocompatibility, drug loading capacities and promising release properties [84,85,86,87,88]. Hydrogels are usually divided into two classes based on their network structures, which are formed by either covalent crosslinking or by various non-covalent interactions (Figure 6). Chemical gels possess high stability due to their covalent bonds, while the physical gels have relatively low stability due to their reliance on non-covalent interactions [89,90,91,92,93,94,95,96,97]. While they may be less stable than chemical gels, physical gels may have the ability to exhibit stimuli-responsive behavior or to undergo reversible changes in response to their surrounding environments. The formation mechanisms, characteristics and biomedical applications of the supramolecular hydrogels, both chemical gels and physical gels, are listed in Table 1.

### 4.1. CD/PPO-b-PEO-b-PPO

Nie *et al.* [98] reported their research on the hydrogel formation of PPO-*b*-PEO-*b*-PPO triblock copolymers with α-CD in aqueous solutions. In particular, using phase diagrams and DSC analysis, they investigated the sol-gel phase transitions and attempted to explain the gelation kinetics and the gel rheological properties of these systems. After the addition of the α-CD, the driving force of the gelation process came from the combination of α-CD and the PEO blocks of the PPO-*b*-PEO-*b*-PPO copolymer. Furthermore, the gel for their characters of thixotropic and reversible would be a high-efficiency drug-delivery system if used as an injectable gel.

In comparison with the inclusion complex formation of CD/PPO-*b*-PEO-*b*-PPO, Park *et al.* [99] developed a buccal-paclitaxel delivery system that was accomplished simply by the addition to PF127 and PEO in the inclusion complex of paclitaxel and (2,6-di-*O*-methyl)-β-CD(DM-β-CD). In this system, the paclitaxel was incorporated into DM-β-CD to form the gel to improve its aqueous solubility while the introduction of PF127 and PEO increased the thermoresponsive and mucoadhesive properties. More importantly, the paclitaxel gel exhibits a sol-gel transition property, and it thus has potential a pplications for a delivery system of anticancer drugs. In particular, this sol-gel transition could be employed to achieve triggered release in response to the external conditions encountered at a tumor site.

Schlatter *et al.* [100] reported the kinetics of the gelation and relative contributions of α-CD and PEO to the cohesion of CD/PEO physical gels. They found that this gel exhibited the non-monotonous evolution behavior by differential scanning calorimetry measurements in concentrated DMSO. In their data, two distinct endothermic peaks, at 29.4 and 32.0 °C, were found in the measurements of these physical gels, which were attributed to the crystals of naked PEO segments and the aggregation of α-CD, respectively.

### 4.2. Poly-α-CD/PEO

As has been described previously, α-CDs have the ability to thread along PEO chains to form PPRs or PRs [84,85,86,87]. However, the complexes formed by poly-α-CD and PEO remain relatively unexplored. Alvarez-Lorenzo *et al.* [101] prepared an injectable supramolecular gel of poly-α-CD and PEO-based copolymers for controlled drug release. The content of α-CD in the composition of the poly-α-CD polymers was greater than 53%, characterized by NMR and the molecular weight evaluated using static light scattering (SLS) was found to be about 455,000 ± 12,000 g·mol^−1^. Supramolecular assemblies occurred by mixing poly-α-CD (20%–40% *w*/*v*) with a PEO-based polymer (*i.e.*, PEG, Pluronic^®^ F127 or Tetronic^®^ 908) (10%–15% *w*/*v*). The gel was formed in less than 24 h if the concentration of poly-α-CD solution and PEO-based copolymer dispersion reached 40% and 10%, respectively. The drugs were easy to load onto any of the polymer dispersions, by only pouring with a syringe that was fitted with an 18G needle, because of their dynamic assembly character of poly-α-CD with the PEO-based copolymers. CD-polymers, in comparison with pristine CDs, have many unique properties, such as the greater aqueous solubility and higher stability [102,103]. In particular, the CD-polymers would form gel or nanocrystal [104,105] in the presence of hydrophilic polymers with complementary substituents [106,107].The 3D-supramolecular gels were formed by the packing of α-CD units of poly-α-CD threading along the PEO blocks, in which water-soluble poly-α-CDs were synthesized by crosslinking α-CD with epichlorohydrin in alkaline medium [101]. The 3D-poly(pseudo)rotaxane gels with excellent cytocompatibility, stability and their sustained drug release would be the novel systems capable of using syringes for the treatment of various diseases.

### 4.3. CD/PEO-b-PVA/PBA

Li *et al.* [108] presented a glucose-responsive hydrogel system. These hydrogels are formed by three components:a poly(ethylene oxide)-*b*-poly (vinyl alcohol) (PEO-*b*-PVA) diblock polymer, α-CD and a phenylboronic acid (PBA)-terminated PEO crosslinker. The hydrogel network was formed via the cooperative interaction forces of the dynamic covalent bonds and inclusion occurred, between PVA and PBA, as well as between PEO and α-CD. Upon the addition of the glucose and other competing polyols, the poly vinyl alcohol (PVA) segment was replaced from the hydrogel system. This glucose-responsive system may be designed as a biosensor to detect or quantify the presence of glucose or fructose and may be used for insulin delivery applications.

### 4.4. CD/PEO

Gaining a firm understanding of the mechanism of drug release is a key requirement for the development of effective drug carriers. Loh *et al.* [109] prepared α-CD/PEO pseudorotaxane hydrogels and loaded them with proteins having different molecular weights. They discussed the influence of various factors on the release, such as the diffusion, erosion or possibly a combination of these two factors as well as the duration of protein release to investigate the drug release mechanism of α-CD-PEO hydrogels. Among various factors such as the PEO concentration, protein concentration, exposed surface area of the gels and so on, they concluded that the surface erosion was the main factor influencing the release of the proteins.

In their investigation of delivery systems based on hydrophilic polymers, Quaglia *et al.* [110] discovered that CDs could affect drug-release behavior in aqueous media and transport properties through lipophilic barriers. In their experiment, hydroxypropyl-β-cyclodextrin (HP-β-CD) was incorporated in a hydrated gel matrix as an aid-excipient. As a result of the introduction of HP-β-CD in erodible hydrophilic tablets, the release rate of two forms increased; whether HP-β-CD was present or absent, Diclofenac was incorporated in PEO matrices in different conditions, such as acid (DicH) or free sodium salt (DicNa). At the same time, the release rate through silicon membranes and porcine buccalmucosa was increased for DicH and decreased for DicNa in the presence of CD, which modified the transport resistance inside the gel matrix. Thus, CDs may serve as release and permeation modulators in hydrophilic matrices and thus may be potentially used to optimize the dissolution and release profile of an oral drug.

**Figure 7:**
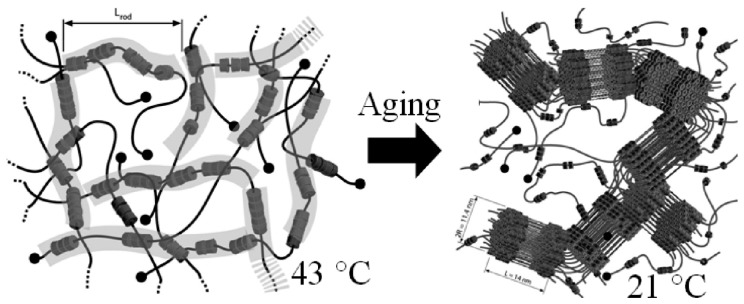
Schematic representation of the temperature-dependent structure of PRs based on CDs at 21 and 43 °C in DMSO. Reprinted with permission from [111]. Copyright (2010) American Chemical Society.

Schlatter *et al.* [111] investigated the kinetics and multiblock copolymer assembly behavior of α-CD/PEO-based polyrotaxanes in dimethyl sulfoxide (DMSO) and they found that the structure of PRs based on CDs were temperature-dependent. At 43 °C, the PEO and PR solutions all remained as liquids, although the PRs presented the multiblock behavior for two types of blocks in DMSO. One type of block was rigid forthe rod like tubes of α-CD, while the other was flexible for the naked PEO segments. When the temperature was cooled down to 21 °C, the PR mixtures gradually formed transparent physical gels. In the PEO/DMSO system, gelation proceeded by the gathering of small nanoscale crystallites and their multiblock copolymer behavior leed to the formation of their regular bundled structures, as shown in Figure 7.

### 4.5. CD/PEO-b-PPO

Hydrogels based on PEO-*b*-PPO block copolymers have great potential as drug delivery systems due to their high solubilization capacity and biocompatibility. Isasi *et al.* [112] investigated two types of hydrogel matrices to evaluate and compare the dissolution profiles of drug. The gels were synthesized by the complexation of Tetronic 90R4 with α-CD in aqueous solution. Meanwhile tablets with similar components as the gels were prepared by freeze-drying. They discovered that the matrix composition, stirring speed, and the nature of the molecule influenced the release profiles of the drugs.

### 4.6. CD/PEO-b-PCL

Li *et al.* [113] prepared novel supramolecular hydrogels based on the diblock copolymer poly(ethylene oxide)-*b*-poly(ε-caprolactone) (PEO-*b*-PCL) and α-CD. They improved the sustained release properties by adjusting the α-CD contents and by substituting part of the PEO with PCL. By investigating the release kinetics of the hydrogels *in vitro*, they found the sustained release character of this supramolecular hydrogel was increased significantly due to the strong hydrophobic interaction of the uncovered PCL blocks in the supramolecular hydrogels. In other words, when the α-CD content was decreased, the release rate was increased.

### 4.7. CD/PEO-PPO Octablock Star Copolymers

Tetronics or poloxamines are four armed copolymers that contain both a PEO block and a PPO block in one arm. When the PEO blocks are on the outside, the Tetronics are named “normal” or “direct”, whereas when the PEO blocks are on the inside, they are referred to as reverse Tetronics. Isasi *et al.* [114] discussed the dissolution profiles of reverse poloxamine/α-CD matrices *in vitro*. They prepared two types of gels based on octablock star polyethylene oxide/polypropylene oxide copolymers (Tetronic 90R4) along with α-CD for use as matrices for drug release. The erosion kinetics of the two different types of matrices, as gels and tablets, were investigated in aqueous media using l-tryptophan (Trp)and bovine serum albumin (BSA) as the release molecules. In addition, by varying the poloxamine/α-CD ratios, the physical properties of poloxamine/α-CD gels could be tailored, such as the viscosity and gel-to-sol transition temperatures, and the erosion kinetics of the gel could also be tuned.

## 5. Conclusions and Future Prospects

In this review, we have described the recent advances in supramolecular architectures based on CDs and poly(ethylene oxide) (PEO) for drug delivery. The introduction of CDs into these supramolecular structures can optimize their performance as drug carriers, such as their biocompatibility, flexibility, and stability. As described in this review, a diverse range of assemblies based on CD/PEO materials have been synthesized and investigated, such as poly(pseudo)rotaxanes, vesicles and supramolecular hydrogels. In future studies on supramolecular architectures based on CD/PEO-materials for drug delivery, there are still many questions and challenges that remain, such as those relating to the physicochemical characteristics, clinical translational performance, the pharmacokinetic profiles, and toxicity of these materials. We believe that significant progress will be made in the construction of nanostructures in the near future and new generations of CD/PEO supramolecules for drug delivery vehicles will play an important role in the treatment of diseases.

## Figures and Tables

**Table 1:** Supramolecular hydrogels based on CDs and PEO formation mechanisms, structures and their characteristics and biomedical applications.

Hydrogel (Cross-Linking Method)	Formation Mechanisms	System	Characteristics or Applications
Physical (reversible)gels	(1) Intermolecular forces hydrogen bonding (2) Hydrophobic injectable effect (3) Electrostatic ionic force or intermolecular assemblies (4) Stereo-complexation (5) Complementary binding	(a) CD/PEO-PPO–PEO	Injectable. Buccal-paclitaxel delivery system [98,99,100]
		(b) Poly-α-CD/PEO	Injectable supramolecular gels [101]
		(c) CD/PEO	CD/PRs with rigid block and flexible block type in DMSO; structure is regular bundles structure at 21 °C [109,110,111]
		(d) CD/PEO-PPO	Sustained delivery [112]
		(e) CD/PEO-PCL	Sustained release, lower molecular weight [113]
		(f) CD/PEO-PPO octablock star copolymers	Biocompatible, sustained [114]
Chemical (permanent)gels	(1) Redox reactions (2) Photo-polymerization (3) Click chemistry (4) Michael reactions (5) Schiff’s base reactions, enzymatic reactions(disulfide-forming reactions)	CD/PEO-PVA/PBA	Pharmaceutical fields (e.g., treatment of diabetes) [107]

## References

[1] Augustynek M., Zemanova A., Kubicek J., Penhaker M. (2015). Model of drugs penetration through biological membrane. Adv. Comput. Commun. Eng. Technol..

[2] Azad T.D., Pan J., Connolly I., Remington A., Wilson C., Grant G.A. (2015). Therapeutic strategies to improve drug delivery across the blood-brain barrier. Neurosurg. Focus.

[3] Patel A., Cholkar K., Agrahari V. (2013). Ocular drug delivery systems: An overview. World J. Pharmacol..

[4] Gao Z., Ma T., Zhao E., Docter D., Yang W., Stauber R.H., Gao M. (2016). Pharmacology & therapeutics. Small.

[5] Deeken J.F., Loscher W. (2007). The blood-brain barrier and cancer: Transporters, treatment, and Trojan horses. Clin. Cancer Res..

[6] Zhao Z., Sagare A.P., Ma Q., Halliday M.R., Kong P., Kisler K., Winkler E.A., Ramanathan A., Kanekiyo T., Bu G. (2015). Central role for PICALM in amyloid-β blood-brain barrier transcytosis and clearance. Nat. Neurosci..

[7] Khumar S., Gupta S.K., Sharma P.K. (2012). Recent developments in targeted drug delivery systems for crossing blood-brain barrier: A review. Int. J. Pharm. Pharm. Sci..

[8] Schneider M., Windbergs M., Daum N., Loretzd B., Collnot E.-M., Hansen S., Schaefer U.F., Lehr C.-M. (2013). Crossing biological barriers for advanced drug delivery. Eur. J. Pharm. Biopharm..

[9] Villanueva M.T. (2015). Drug delivery: Get in there!. Nat. Rev. Cancer.

[10] Larsen K.L. (2002). Large cyclodextrins. J. Incl. Phenom. Macrocycl. Chem..

[11] Ueda H. (2002). Physicochemical properties and complex formation abilities of large-ring cyclodextrins. J. Incl. Phenom. Macrocycl. Chem..

[12] Mondjinou Y.A., Hyun S.-H., Xiong M., Collins C.J., Thong P., Thompson D.H. (2015). Impact of mixed β-cyclodextrin ratios on pluronicrotaxanation efficiency and product solubility. ACS Appl. Mater. Interfaces.

[13] Clementine P., Kevin S.J., Lisbeth G., Justin J. (2013). Gelation kinetics and viscoelastic properties of Pluronic and α-cyclodextrin-based pseudopolyrotaxane hydrogels. Biomacromolecules.

[14] Delbianco M., Bharate P., Varela-Aramburu S., Seeberger P.H. (2016). Carbohydrates in supramolecular chemistry. Chem. Rev..

[15] Wang J., Qiu Z., Wang Y., Li L., Guo X., Pham D.T., Lincoln S.F., Prud’homme R.K. (2016). Supramolecular polymer assembly in aqueous solution arising from cyclodextrin host-guest complexation. Beilstein J. Org. Chem..

[16] Zhang S., Bellinger A.M., Glettig D.L., Barman R., Lee Y.A., Zhu J., Cleveland C., Montgomery V.A., Gu L., NashL D. (2015). A pH-responsive supramolecular polymer gel as an enteric elastomer for use in gastric devices. Nat. Mater..

[17] Cao Z., Woortman A.J.J., Rudolf P., Loos K. (2015). Facile synthesis and structural characterization of amylose—Fatty acid inclusion complexes. Macromol. Biosci..

[18] Francisco V., Garcia-Rio L. (2014). Interaction of bolaform surfactants withp-sulfonatocalix[4]arene: The role of two positive charges in the binding. Langmuir.

[19] Vecsernyѐs M., Fenyvesi F., Bacskay I., Deli M.A., Szente L., Fenyvesi É. (2014). Cyclodextrins, bloode-brain barrier, and treatment of neurological diseases. Arch. Med. Res..

[20] Shreyas S., Aniruddh S., Ki-Bum L. (2016). Nanotechnology-based approaches for guiding neural regeneration. Acc. Chem. Res..

[21] Simões S.M.N., Rey-Rico A., Concheiro A., Alvarez-Lorenzo C. (2015). Supramolecular cyclodextrin-based drug nanocarriers. Chem. Commun..

[22] Ma X., Zhao Y. (2015). Biomedical applications of supramolecular systems based on host-guest interactions. Chem. Rev..

[23] Davis M.E., Brewster M.E. (2004). Cyclodextrin-based pharmaceutics: Past, present, and future. Nat. Rev. Drug Discov..

[24] Loftsson T., Brewster M.E. (2011). Pharmaceutical applications of cyclodextrins: Effects on drug permeation through biological membranes. J. Pharm. Pharmacol..

[25] Chu C.W., Cheng C.C., Bastakoti B.P., Kuo S.-W. (2016). Hierarchical mesoporous silicas templated by PE-*b*-PEO-*b*-PLA triblock copolymer for fluorescent drug delivery. RSC Adv..

[26] Serizawa T., Fukuta H., Date T., Sawada T. (2016). Affinity-based release of polymer-binding peptides from hydrogels with the target segments of peptides. Chem. Commun..

[27] Lian Z., Ye L. (2013). Effect of PEO on the network structure of PVA hydrogels prepared by freezing/thawing method. J. Appl. Polym. Sci..

[28] Riess G. (2003). Micellization of block copolymers. Prog. Polym. Sci..

[29] Zhang L., Eisenberg A. (1995). multiple morphologies of “crew-cut” aggregates of polystyrene-*b*-poly(acrylic acid) block copolymers. Science.

[30] Cameron N.S., Corbierre M.K., Eisenberg A.C. (1999). 1998 E.W.R. Steacie Award Lecture Asymmetric amphiphilic block copolymers in solution: A morphological wonderland. J. Chem..

[31] Eisenberg M., Eisenberg A. (2012). Self-assembly of block copolymers. Chem. Soc. Rev..

[32] Harada A., Kamachi M. (1990). Complex formation between cyclodextrin and poly(propylene glycol). J. Chem. Soc. Chem. Commun..

[33] Harada A., Li J., Kamachi M. (1993). Preparation and properties of inclusion complexes of polyethylene glycol with alpha-cyclodextrin. Macromolecules.

[34] Wei Z., Yang J., Zhou J., Xu F., Zrínyi M., Dussault P.H., Osadag Y., Chen Y. (2014). Self-healing gels based on constitutional dynamic chemistry and their potential applications. Chem. Soc. Rev..

[35] Arranja A., Waton G., Schosseler F., Mendesb E. (2016). Lack of a unique kinetic pathway in the growth and decay of Pluronic micelles. Soft Matter.

[36] Mehanny M., Hathout R.M., Geneidi A.S., Mansour S. (2016). Exploring the use of nanocarrier systems to deliver the magical molecule; Curcumin and its derivatives. J. Control. Release.

[37] D’Arcy R., Siani A., Lallana E., Tirelli N. (2015). Influence of primary structure on responsiveness. Oxidative, thermal, and thermo-oxidative responses in polysulfides. Macromolecules.

[38] Gao N., Lü S., Gao C., Wang X., Xu X., Bai X., Feng C., Liu M. (2016). Injectable shell-crosslinked F127 micelle/hydrogel composites with pH and redox sensitivity for combined release of anticancer drugs. Chem. Eng. J..

[39] Lisi R., Lazzara G., Milioto S., Muratore N. (2008). Polystyrene nanoparticles in the presence of (ethylene oxide)_13_(propylene oxide)_30_(ethylene oxide)_13_, *N*,*N*-dimethyloctylamine-*N*-oxide and their mixtures. A calorimetric and dynamic light scattering study. Phys. Chem. Chem. Phys..

[40] Ramírez P., Stocco A., Muñoz J., Miller R. (2012). Interfacial rheology and conformations of triblock copolymers adsorbed onto the water-oil interface. J. Colloid Interface Sci..

[41] Lisi R., Lazzara G., Milioto S., Muratore N. (2006). Volumes of aqueous block copolymers based on poly(propylene oxides) and poly(ethylene oxides) in a large temperature range: A quantitative description. J. Chem. Thermodyn..

[42] Loh W., Somasundaran P. (2006). Encyclopedia of Surface and Colloid Science.

[43] Kong T., Ye L., Zhang A.Y. (2014). Loose-fit polypseudorotaxanes constructed from γ-CDs and PHEMA-PPG-PEG-PPG-PHEMA. Beilstein J. Org. Chem..

[44] Jiang L., Ye L., Zhang A.Y. (2014). Self-assembly of polyrotaxanes synthesized via click chemistry of azido-endcapped PNIPAAm-*b*-Pluronic F68-*b*-PNIPAAm/γ-CD with propargylamine-substituted β-CDs. Macromol. Chem. Phys..

[45] Wang J., Li S., Ye L., Zhang A., Feng Z. (2012). Formation of a polypseudorotaxane via self-assembly of γ-cyclodextrin with poly(*N*-isopropylacrylamide). Macromol. Rapid Commun..

[46] Gao P., Wang J., Wang P.J., Ye L., Zhang A., Feng Z. (2012). Loose-fit polypseudorotaxanes fabricated by γ-CDs threaded onto a single PNIPAAm-PEG-PNIPAAm chain in aqueous solution. Macromol. Chem. Phys..

[47] Harada A., Li J., Kamachi M. (1994). Double-stranded inclusion complexes of cyclodextrin threaded on poly(ethylene glycol). Nature.

[48] Takahashi A., Katoono R., Yui N. (2009). Loose-fit polyrotaxane composed of gamma-cyclodextrin and single poly(ethyelene glycol) chain: Making room in gamma-cd cavity for additional inclusion complexation. Macromolecules.

[49] Parmar A., Yerramilli U., Bahadur P. (2012). Effect of hydrophobicity of PEO-PPO-PEO block copolymers on micellization and solubilization of a model drug nimesulide. J. Surfactants Deterg..

[50] Dreiss C.A., Nwabunwanne E., Liu R., Brooks N.J. (2009). Assembling and de-assembling micelles: Competitive interactions of cyclodextrins and drugs with Pluronics. Soft Matter.

[51] Wang J., Gao P., Ye L., Zhang A., Feng Z. (2010). Solvent- and thermoresponsive polyrotaxanes with β-cyclodextrin dispersed/aggregated structures on a Pluronic F127 backbone. J. Phys. Chem. B.

[52] Lazzara G., Milioto S. (2008). Copolymer-cyclodextrin inclusion complexes in water and in the solid state. A physico-chemical study. J. Phys. Chem. B.

[53] Zhu W., Zhang K., Chen Y. (2014). Block copolymer micelles as carriers of transition metal ions Y(III) and Cu(II) and gelation thereof. Polymer.

[54] Wesley R.D., Cosgrove T., Thomson L., Armes S.P., Billingham N.C., Baines F.L. (2000). Hydrodynamic layer thickness of a polybase brush in the presence of salt. Langmuir.

[55] Cosgrove T., Mears S.J., Obey T., Thompsonb L., Wesleya R.D. (1999). Polymer, particle, surfactant interactions. Colloids Surfaces A Physicochem. Eng. Asp..

[56] Ceccato M., LoNostro P., Baglioni P. (1997). α-Cyclodextrin/Polyethylene glycol polyrotaxane: A study of the threading process. Langmuir.

[57] Joseph J., Dreiss C.A., Cosgrove T. (2008). Stretching a polymer brush by making *in situ* cyclodextrin inclusion complexes. Langmuir.

[58] Travelet C., Schlatter G., Hebraud P., Brochon C., Lapp A., Hadziioannou G. (2009). Formation and self-organization kinetics of α-CD/PEO-based pseudo-polyrotaxanes in water. A specific behavior at 30 °C. Langmuir.

[59] Luis N., Eduardo S., José L., Francisco J.O. (2012). Competitive displacement of drugs from cyclodextrin inclusion complex by polypseudorotaxane formation with poloxamer: Implications in drug solubilization and delivery. Eur. J. Pharm. Biopharm..

[60] Larrañeta E., Isasi J.R. (2012). Self-assembled supramolecular gels of reverse poloxamers and cyclodextrins. Langmuir.

[61] Yang C., Ni X., Li J. (2009). Synthesis of polyrotaxanes consisting of multiple a-cyclodextrin rings threaded on reverse Pluronic PPO-PEO-PPO triblock copolymers based on block-selected inclusion complexation. Eur. Polym. J..

[62] Iguchi H., Uchida S., Koyama Y., Takata T. (2013). Polyester-containing α-cyclodextrin-based polyrotaxane: Synthesis by living ring-opening polymerization, polypseudorotaxanation, and end capping using nitrile *N*-oxide. ACS Macro Lett..

[63] Stayton P.S., Shimoboji T., Long C., Chilkoti A., Ghen G., Harris J.M., Hoffman A.S. (1995). Control of protein-ligand recognition using a stimuli-responsive polymer. Nature.

[64] Chen G.H., Hoffman A.S. (1995). Graft copolymers that exhibit temperature-induced phase transitions over a wide range of pH. Nature.

[65] Yang C., Li J. (2009). Thermoresponsive behavior of cationic polyrotaxane composed of multiple pentaethylenehexamine-graftedα-cyclodextrins threaded on poly(propyleneoxide)-poly(ethylene oxide)-poly(propylene oxide) triblock copolymer. J. Phys. Chem. B.

[66] Discher D.E., Eisenberg A. (2002). Polymer Vesicles. Science.

[67] Meng F., Zhong Z., Feijen J. (2009). Stimuli-responsive polymersomes for programmed drug delivery. Biomacromolecules.

[68] Jiang W., Zhou Y., Yana D. (2015). Hyperbranched polymer vesicles: From self-assembly, characterization, mechanisms, and properties to applications. Chem. Soc. Rev..

[69] Zhou Y., Yan D. (2005). Real-time membrane fusion of giant polymer vesicles. J. Am. Chem. Soc..

[70] Chen J., Liu Q., Xiao J. (2015). EpCAM-Antibody-labeled noncytotoxic polymer vesicles for cancer stem cells-targeted delivery of anticancer drug and siRNA. Biomacromolecules.

[71] Gu W., Li Q., Lu H., Fang L., Chen Q., Yang Y., Gao H. (2015). Construction of stable polymeric vesicles based on azobenzene and beta-cyclodextrin grafted poly(glycerol methacrylate)s for potential applications in colon-specific drug delivery. Chem. Commun..

[72] Palivan C.G., Goers R., Najer A., Zhang X., Cara A., Meier W. (2016). Bioinspired polymer vesicles and membranes for biological and medical applications. Chem. Soc. Rev..

[73] Wang J., Ni Y., Jiang W., Li H., Liu Y., Lin S., Zhou Y., Yan D. (2015). Self-crosslinking and surface-engineered polymer vesicles. Small.

[74] Schulz M., Binder W.H. (2015). Mixed hybrid lipid/polymer vesicles as a novel membrane platform. Macromol. Rapid Commun..

[75] Ding J., Chen L., Xiao C., Chen L., Zhuang X., Chen X. (2014). Noncovalent interaction-assisted polymeric micelles for controlled drug delivery. Chem. Commun..

[76] Du J., Liu Q. (2016). Multifunctional polymer vesicles for cancer stem cells-targeted drug/siRNA therapy. Cancer Cell Microenviron..

[77] Robbins G.P., Saunders R.L., Haun J.B., Rawson J., Therien M.J., Hammer D.A. (2010). Tunable leuko-polymersomes that adhere specifically to inflammatory markers. Langmuir.

[78] Zhang Z., Ding J., Chen X., Xiao C., He C., Zhuang X., Chen L., Chen X. (2013). Intracellular pH-sensitive supramolecular amphiphiles based on host-guest recognition between benzimidazole and beta-cyclodextrin as potential drug delivery vehicles. Polym. Chem..

[79] Jin H.B., Zhou Y.F., Huang W., Yan D. (2010). Polymerization-like multilevel hierarchical self-assembly of polymer vesicles into macroscopic superstructures with controlled complexity. Langmuir.

[80] Smart T.P., Fernyhough C., Ryan A.J., Battaglia G. (2008). Controlling fusion and aggregation in polymersome dispersions. Macromol. Rapid Commun..

[81] Liu G., Jin Q., Liu X., Lv L., Chen C., Ji J. (2011). Biocompatible vesicles based on PEO-*b*-PMPC/a-cyclodextrin inclusion complexes for drug delivery. Soft Matter.

[82] Jin Q., Liu G., Ji J. (2014). Supramolecular micelles and reverse micelles based on cyclodextrinpolyrotaxanes. Chin. J. Chem..

[83] Jin H., Liu Y., Zheng Y., Huang W., Zhou Y., Yan D. (2012). Cytomimetic large-scale vesicle aggregation and fusion based on host-guest interaction. Langmuir.

[84] Bajpai A.K., Shukla S.K., Bhanu S., Kankane S. (2008). Responsive Polymers in controlled drug delivery. Prog. Polym. Sci..

[85] Puppi D., Chiellini F., Piras A.M., Chiellini E. (2010). Polymeric materials for bone and cartilage repair. Prog. Polym. Sci..

[86] Hoffman A.S. (2002). Hydrogels for biomedical applications. Adv. Drug Deliv. Rev..

[87] Zhang J., Ma P.X. (2013). Cyclodextrin-based supramolecular systems for drug delivery: Recent progress and future perspective. Ad. Drug Deliv. Rev..

[88] Fu C., Lin X., Wang J., Zheng X., Li X., Lin Z., Lin G. (2016). Injectable micellar supramolecular hydrogel for delivery of hydrophobic anticancer drugs. J. Mater. Scie.Mater. Med..

[89] De France K.J., Chan K.J.W., Cranston E.D., Hoare T. (2016). Enhanced mechanical properties in cellulose nanocrystal–poly(oligoethylene glycol methacrylate) injectable nanocomposite hydrogels through control of physical and chemical cross-linking. Biomacromolecules.

[90] Alexandridis P., Hatton T.A. (1995). Poly(ethylene oxide)-poly(pro-pylene oxide)-poly(ethylene oxide) block copolymer surfactants in aqueous solutions and at interfaces: Thermodynamics, structure, dynamics, and modeling. Colloids Surf. A Physicochem. Eng. Asp..

[91] Oberoi H.S., Yorgensen Y.M., Morasse A., Evans J.T., Burkhart D.J. (2016). PEG modified liposomes containing CRX-601 adjuvant in combination with methylglycol chitosan enhance the murine sublingual immune response to influenza vaccination. J. Control. Release.

[92] Bin Imran A., Esaki K., Gotoh H., Seki T., Ito K., Sakai Y., Takeoka Y. (2014). Extremely stretchable thermosensitive hydrogels by introducing slide-ring polyrotaxane cross-linkers and ionic groups into the polymer network. Nat. Commun..

[93] Zhao P., Zheng M., Luo Z., Gong P., Gao G., Sheng Z., Zheng C., Ma Y., Cai L. (2015). NIR-driven smart theranostic nanomedicine for on-demand drug release and synergistic antitumour therapy. Nat. Sci. Rep..

[94] Jeong B., Bae Y.H., Kim S.W. (2000). Drug release from biodegradable injectable thermosensitive hydrogel of PEG-PLGA-PEG tri-block copolymers. J. Control. Release.

[95] Song G., Zhang L., He C., Fang D., Whitten P.G., Wang H. (2013). Facile fabrication of tough hydrogels physically cross-linked by strong cooperative hydrogen bonding. Macromolecules.

[96] De Jong S.J., De Smedt S.C., Demeester J., van Nostrum C.F., Kettenes-van den Bosch J.J., Hennink W.E. (2001). Biodegradable hydrogels based on stereo complex formation between lactic acid oligomers grafted to dextran. J. Control. Release.

[97] Appel E.A., Tibbitt M.W., Webber M.J., Mattix B.A., Veiseh O., Langer R. (2015). Self-assembled hydrogels utilizing polymer-nanoparticle interactions. Nat. Commun..

[98] Ni X., Cheng A., Li J. (2009). Supramolecular hydrogels based on self-assembly between PEO-PPO-PEO triblock copolymers and α-cyclodextrin. J. Biomed. Mater. Res. Part A.

[99] Choi S., Lee S., Kang B. (2014). Thermosensitive and mucoadhesive sol-gel composites of paclitaxel/dimethyl-β-cyclodextrin for buccal delivery. PLoS ONE.

[100] Travelet C., Schlatter G., Hebraud P., Brochon C., Anokhin D.V., Ivanov D.A., Hadziioannou G. (2010). Physical gels based on polyrotaxanes: Kinetics of the gelation, and relative contributions of α-cyclodextrin and poly(ethylene oxide) to the gel cohesion. Macromol. Symp..

[101] Simões S., Veiga F., Ribeiro A.C.F., Figueiras A.R., Taboada P., Concheiro A., Alvarez-Lorenzo C. (2014). Supramolecular gels of poly-α-cyclodextrin and PEO-based copolymers for controlled drug release. Eur. J. Pharm. Biopharm..

[102] Danel C., Azaroual N., Chavaria C. (2013). Comparativestudy of the complex forming ability and enantioselectivity of cyclodextrin polymers by CE and ^1^H NMR. Carbohydr. Polym..

[103] Concheiro A., Alvarez-Lorenzo C. (2013). Chemically cross-linked and grafted cyclodextrin hydrogels: From nanostructures to drug-eluting medical devices. Adv. Drug Deliv. Rev..

[104] Koopmans C., Ritter H. (2008). Formation of physical hydrogels via host guest interactions of β-cyclodextrin polymers and copolymers bearing adamantly groups. Macromolecules.

[105] Nielsen A.L., Steffensen K., Larsen K.L. (2009). Self-assembling microparticles with controllable disruption properties based on cyclodextrin interactions. Colloids Surf. B Biointerfaces.

[106] Gref R., Amiel C., Molinard K., Daoud-Mahammed S., Sébille B., Gillet B., Beloeil J., Ringard C., Rosilio V., Poupaert J. (2006). New self-assembled nanogels based on host-guest interactions: Characterization and drug loading. J. Control. Release.

[107] Daoud-Mahammed S., Ringard-Lefebvre C., Razzouq N. (2007). Spontaneous association of hydrophobized dextran and poly-β-cyclodextrin into nanoassemblies: Formation and interaction with a hydrophobic drug. J. Colloid Interface Sci..

[108] Yang T., Ji R., Deng X. (2014). Glucose-responsive hydrogels based on dynamic covalent chemistry and inclusion complexation. Soft Matter.

[109] Chee P., Prasad A., Fang X. (2014). Supramolecular cyclodextrin pseudorotaxane hydrogels: A candidate for sustained release?. Mater. Sci. Eng. C.

[110] Miro A., Rondinone A., Nappi A. (2009). Modulation of release rate and barrier transport of Diclofenac incorporated in hydrophilic matrices: Role of cyclodextrins and implications in oral drug delivery. Eur. J. Pharm. Biopharm..

[111] Travelet C., Hebraud P., Perry C. (2010). Temperature-dependent structure of γ-CD/PEO-based polyrotaxanes in concentrated solution in DMSO: Kinetics and multiblock copolymer behavior. Macromolecules.

[112] Larrañeta E., Arriz C., Velaz I., Zornoza A., Machín R., Isasi J. (2014). *In vitro* release from reverse poloxamine/α-cyclodextrin matrices: Modelling and comparison of dissolution profiles. J. Pharm. Sci..

[113] Li X., Li J. (2008). Supramolecular hydrogels based on inclusion complexation between poly(ethylene oxide)-β-poly(ɛ-caprolactone) diblock copolymer and α-cyclodextrin and their controlled release property. J. Biomed. Mater. Res. Part A.

[114] Larrañeta E., Isasi J. (2014). Non-covalent hydrogels of cyclodextrins and poloxamines for the controlled release of proteins. Carbohydr. Polym..

